# Ionic Liquid-Assisted Sequential Ultrasound–Microwave Extraction of Monoterpene Glycosides from *Radix Paeoniae Alba*: Multi-Marker Optimization, UPLC-QTOF-MS Profiling and Molecular Interaction Insights

**DOI:** 10.3390/molecules31132342

**Published:** 2026-07-03

**Authors:** Jiachen Shen, Jieru Zhang, Xiaoming Peng, Ying Yang

**Affiliations:** College of New Materials and Chemical Engineering, Beijing Institute of Petrochemical Technology, Beijing 102617, China; 2024520079@bipt.edu.cn (J.S.);

**Keywords:** *Radix Paeoniae Alba*, paeoniflorin, oxypaeoniflorin, albiflorin, ionic liquid, sequential ultrasound–microwave extraction, UPLC-QTOF-MS, response surface methodology, molecular interaction insights

## Abstract

*Radix Paeoniae Alba*, the dried root of *Paeonia lactiflora* Pall., contains characteristic monoterpene glycosides, but efficient recovery of these polar constituents remains challenging. This study developed an ionic liquid-assisted sequential ultrasound–microwave extraction method and evaluated paeoniflorin, oxypaeoniflorin and albiflorin by HPLC as multi-marker responses. Among the ionic liquids tested, 1-propyl-3-methylimidazolium dihydrogen phosphate showed the best extraction response. Box–Behnken response surface optimization gave practical extraction conditions of a solid-to-liquid ratio of 1:26 g/mL, ionic liquid concentration of 0.12 mol/L and ultrasound time of 22 min. Under these conditions, paeoniflorin and total marker glycosides reached 29.12 and 34.98 mg/g dry material, respectively, representing increases of 32.4% and 34.5% compared with conventional reflux extraction. UPLC-QTOF-MS profiling provided complementary chemical profile information for the optimized extract and tentatively annotated Paeonia-related monoterpene glycoside derivatives, galloylated glucose derivatives and polyphenolic constituents. Electrostatic potential, SAPT and non-covalent interaction analyses, supported by ^1^H NMR chemical shift perturbation, suggested possible hydrogen bonding, electrostatic and dispersion interactions between paeoniflorin and the selected ionic liquid. These results support the optimized process as an efficient extraction approach and provide molecular interaction insights into ionic liquid-assisted recovery of monoterpene glycosides.

## 1. Introduction

*Radix Paeoniae Alba*, the dried root of *Paeonia lactiflora* Pall., is a widely used medicinal material in traditional Chinese medicine. It has been applied in various clinical and traditional formulations and is generally recognized for its multiple pharmacological properties. Previous studies have shown that monoterpene glycosides, tannins, phenolic acids and other polar constituents are important chemical groups in *Radix Paeoniae Alba* and related Paeonia materials [1,2]. Among these constituents, paeoniflorin, albiflorin and oxypaeoniflorin have been widely used as representative marker compounds in analytical, pharmacokinetic and quality control studies of *Radix Paeoniae Alba* [2,3,4].

Paeoniflorin, albiflorin and oxypaeoniflorin are representative monoterpene glycoside markers of *Radix Paeoniae Alba*. Structurally, these compounds contain sugar-derived moieties and multiple oxygen-containing functional groups, such as hydroxyl, ether and ester groups, which are closely related to their polarity and extraction behavior. Therefore, a multi-marker strategy based on paeoniflorin, oxypaeoniflorin, albiflorin and their summed content is more informative than evaluating paeoniflorin alone. The present study focused on these representative and analytically accessible monoterpene glycoside markers, rather than attempting to classify all possible mono-, di- or tri-glycosylated metabolites in the plant matrix.

Efficient extraction is a key step for the utilization, quality control and product development of medicinal plant resources. Conventional extraction methods such as reflux extraction are simple and widely used, but they often require relatively long extraction times and may show limited extraction efficiency. Recent green extraction research has shifted from simple replacement of organic solvents toward the integration of solvent design, process intensification and mechanistic understanding [5]. Deep eutectic solvents and natural deep eutectic solvents have attracted increasing attention because their hydrogen bond networks, tunable polarity and adjustable water content can be used to extract diverse plant-derived bioactive compounds [6,7,8]. Nevertheless, solvent viscosity, target release, solvent recovery and process compatibility remain important considerations when applying DES/NADES systems to phytochemical extraction.

Alongside DES/NADES, ionic liquids remain important designer solvents for natural product extraction. Their cations, anions and alkyl chains can be systematically varied, allowing structure–performance relationships to be explored more directly than in many multicomponent eutectic systems [9,10,11,12]. In this work, imidazolium-based ionic liquids were used as structurally tunable extraction additives to examine how anion type and alkyl chain length affect the recovery of representative Paeonia monoterpene glycosides under sequential ultrasound–microwave-assisted conditions.

Process intensification was also considered in this work. Ultrasound-assisted extraction can facilitate solvent penetration, matrix disruption and mass transfer through cavitation and mechanical effects, whereas microwave heating can accelerate solvent heating and molecular diffusion [13,14]. A sequential ultrasound–microwave process may therefore improve the recovery of polar constituents from plant matrices. However, extraction optimization based only on response values cannot fully explain the chemical context of the resulting extract or the possible molecular interactions between the target compounds and the selected solvent.

For this reason, targeted HPLC quantification and non-targeted UPLC-QTOF-MS profiling were combined in the present study. HPLC was used to quantify paeoniflorin, oxypaeoniflorin and albiflorin as marker responses for extraction optimization, whereas UPLC-QTOF-MS was used to provide a broader chemical profile of the optimized extract and to tentatively annotate representative Paeonia-related constituents [15,16].

In this study, an ionic liquid-assisted sequential ultrasound–microwave extraction process was developed for monoterpene glycosides from *Radix Paeoniae Alba*. The extraction process was optimized through ionic liquid screening, single-factor experiments and response surface methodology. UPLC-QTOF-MS was further applied to obtain a chemical profile of the optimized extract. Finally, electrostatic potential analysis, SAPT energy decomposition, non-covalent interaction visualization and ^1^H NMR chemical shift perturbation were used to provide supportive molecular-level evidence for possible interactions between paeoniflorin and the selected ionic liquid [17,18,19,20,21]. This study aimed to establish an efficient multi-marker extraction strategy and to provide a plausible molecular interaction explanation for ionic liquid-assisted extraction, without overextending the simplified molecular model to the full complexity of the plant matrix.

## 2. Results and Discussion

### 2.1. HPLC Method Validation for Three Marker Monoterpene Glycosides

An HPLC method was established for the simultaneous determination of three representative monoterpene glycosides in *Radix Paeoniae Alba*, namely, oxypaeoniflorin, albiflorin and paeoniflorin. Under the established chromatographic conditions, oxypaeoniflorin was detected at 258 nm, whereas albiflorin and paeoniflorin were detected at 230 nm. The method was evaluated in terms of selectivity, linearity, precision, repeatability, sensitivity and recovery-based trueness.

**Figure 1:**
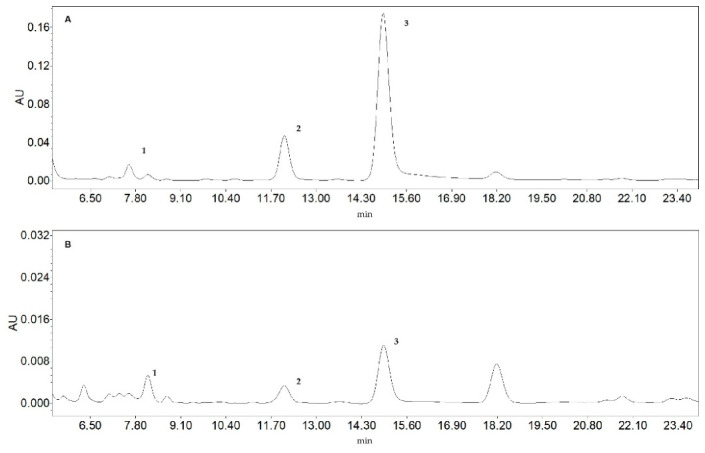
Representative HPLC chromatograms of the optimized ionic liquid-assisted sequential ultrasound–microwave extract of *Radix Paeoniae Alba*. (**A**) Optimized extract detected at 230 nm; (**B**) optimized extract detected at 258 nm. Peaks: 1, oxypaeoniflorin; 2, albiflorin; 3, paeoniflorin. Mixed standard chromatograms and additional spiked-sample chromatograms are provided in Figure S1 for selectivity evaluation.

Representative HPLC chromatograms of the optimized extract are shown in Figure 1, while the mixed standard chromatograms and additional spiked-sample chromatograms for selectivity evaluation are provided in Figure S1. The three marker compounds showed distinguishable chromatographic peaks under the established conditions. No visually observable co-eluting interference was found at the retention times of oxypaeoniflorin, albiflorin or paeoniflorin in the optimized extract. The supplementary standard and spiked-sample chromatograms further supported the applicability of the established HPLC method for the present comparative extraction study.

The validation parameters are summarized in Table 1. The retention times of oxypaeoniflorin, albiflorin and paeoniflorin were 6.351, 12.135 and 14.995 min, respectively. The resolution between oxypaeoniflorin and its adjacent peak was 1.599, which was close to the commonly used minimum criterion. Therefore, the chromatographic region around oxypaeoniflorin was further checked using the optimized extract and spiked extract chromatograms. Although the resolution was close to the criterion, no obvious co-eluting peak was observed at the oxypaeoniflorin retention time under the established conditions. The theoretical plate numbers were higher than 6500, and the tailing factors ranged from 1.066 to 1.121, indicating acceptable chromatographic efficiency and peak symmetry.

Good linearity was obtained over the injected-amount range of 0.0391–2.50 μg, with correlation coefficients above 0.999. The LOD values of oxypaeoniflorin, albiflorin and paeoniflorin were 3.91, 1.95 and 1.95 μg/mL, respectively, and the corresponding LOQ values were 13.03, 6.50 and 6.50 μg/mL. The precision and repeatability RSD values were below 2.0%, indicating acceptable instrumental precision and repeatability for comparative quantification.

Recovery experiments were used to evaluate recovery-based trueness. The mean recoveries of oxypaeoniflorin, albiflorin and paeoniflorin were 101.6%, 101.0% and 101.5%, respectively. These slightly above 100% values may be associated with minor matrix effects, baseline integration variation or sample preparation variability, but they remained acceptable for comparative quantification of the three marker monoterpene glycosides in the present extraction study.

In this study, the total marker monoterpene glycoside content was calculated as the sum of oxypaeoniflorin, albiflorin and paeoniflorin and expressed as mg/g dry material. This multi-marker evaluation was used to better reflect the recovery of representative monoterpene glycosides from *Radix Paeoniae Alba*.

### 2.2. Comparison of Conventional Reflux Extraction and Sequential Ultrasound–Microwave-Assisted Extraction

**Figure 2:**
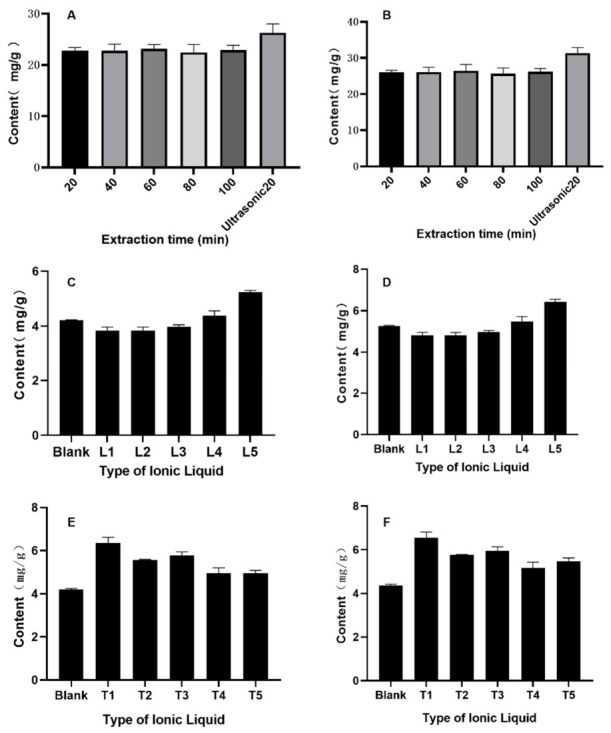
Comparison of extraction methods and screening of ionic liquids for monoterpene glycoside extraction from *Radix Paeoniae Alba*. (**A**) Effect of extraction method on paeoniflorin content; (**B**) effect of extraction method on total marker monoterpene glycoside content; (**C**) effect of anion type on paeoniflorin content; (**D**) effect of anion type on total marker monoterpene glycoside content; (**E**) effect of alkyl chain length on paeoniflorin content; (**F**) effect of alkyl chain length on total marker monoterpene glycoside content. Blank represents the extraction system without ionic liquid. L1–L5 and T1–T5 correspond to the ionic liquids listed in Supplementary Table S2. Data are expressed as mean ± SD (n = 3).

To evaluate the feasibility of introducing an intensified extraction process, conventional reflux extraction was compared with sequential ultrasound–microwave-assisted extraction under comparable solvent conditions. For conventional reflux extraction, a 70% ethanol–water solution (*v*/*v*) was used as the extraction solvent. Paeoniflorin content and total marker monoterpene glycoside content were used as the evaluation indexes. Figure 2A,B presents the comparison between conventional reflux extraction and sequential ultrasound–microwave-assisted extraction.

As shown in Figure 2A,B, the paeoniflorin content obtained by reflux extraction remained relatively stable at approximately 22 mg/g dry material when the extraction time was extended from 20 to 100 min. Similarly, the total marker monoterpene glycoside content remained at approximately 26 mg/g dry material. These results indicated that conventional reflux extraction reached an apparent extraction plateau under the tested conditions, and prolonging the reflux time did not markedly increase the recovery of the target compounds.

In contrast, the sequential ultrasound–microwave-assisted process achieved higher extraction responses within a shorter processing time. After ultrasound treatment for 20 min followed by microwave treatment for 10 min, the paeoniflorin content and total marker monoterpene glycoside content reached 26.29 and 31.39 mg/g dry material, respectively. Compared with conventional reflux extraction at 80 min, these values increased by 19.5% and 20.7%, respectively.

The improvement may be attributed to process intensification during the sequential treatment. Ultrasound treatment can promote solvent penetration and matrix disruption through cavitation and mechanical effects, whereas microwave heating can accelerate solvent heating and molecular diffusion. However, ultrasound-only and microwave-only experiments were not included in the original design. Therefore, the individual contributions of ultrasound and microwave treatment cannot be quantitatively separated in this study, and the observed improvement should be interpreted as the process-level enhancement of the complete sequential ultrasound–microwave-assisted extraction procedure rather than as proof of a true synergistic effect.

### 2.3. Effect of Ionic Liquid Structure on Extraction Response

After confirming the feasibility of the sequential ultrasound–microwave-assisted extraction process, ionic liquids were further screened as extraction additives. The screening was designed as an exploratory structure–response comparison rather than as a complete physicochemical optimization of ionic liquids. Two structural factors were considered: anion type and cationic alkyl chain length. Paeoniflorin content and total marker monoterpene glycoside content were used as the response values.

First, imidazolium-based ionic liquids with different anions were compared under the same extraction conditions. As shown in Figure 2C,D, the extraction responses varied among the tested anions. Among them, the dihydrogen phosphate-containing ionic liquid gave the highest paeoniflorin content and total marker monoterpene glycoside content. This result suggested that anion structure influenced the extraction of polar monoterpene glycosides under the tested conditions.

The better extraction response of the dihydrogen phosphate-containing ionic liquid may be related to its ability to interact with polar oxygen-containing functional groups in monoterpene glycosides. Paeoniflorin, oxypaeoniflorin and albiflorin contain multiple hydroxyl, ether and ester groups, which may interact with ionic liquid components through hydrogen bonding, electrostatic and other non-covalent interactions. This trend is consistent with a structure-dependent extraction response, although direct evidence for hydrogen bond formation during extraction was not obtained.

After the anion screening, imidazolium dihydrogen phosphate ionic liquids with different alkyl chain lengths were further compared. As shown in Figure 2E,F, the extraction responses were also affected by alkyl chain length. The 1-propyl-3-methylimidazolium dihydrogen phosphate system showed the highest extraction response among the tested chain-length series and was therefore selected for subsequent single-factor and response surface optimization experiments.

The chain-length-dependent trend may reflect a balance between interaction capacity and mass-transfer behavior. A short alkyl chain may provide an insufficient hydrophobic or dispersion contribution, whereas an excessively long alkyl chain may affect solvent compatibility and mass transfer in the hydroethanolic extraction system. Nevertheless, because viscosity, polarity, conductivity and pH were not independently measured in the present screening experiment, no quantitative relationship between these physicochemical properties and extraction performance can be established. The ionic liquid screening results should therefore be interpreted as empirical structure–extraction response trends within the tested ionic liquid series.

### 2.4. Effects of Extraction Variables on Monoterpene Glycoside Recovery

Single-factor experiments were performed to evaluate the influence of extraction variables on the recovery of paeoniflorin and total marker monoterpene glycosides from *Radix Paeoniae Alba*. The investigated variables included ionic liquid concentration, ultrasonic time, ultrasonic power, solid-to-liquid ratio and microwave temperature. These experiments were conducted under predefined initial conditions and were used mainly to identify influential factors and suitable experimental ranges for subsequent response surface optimization. Therefore, the absolute contents obtained in the single-factor experiments should not be directly compared with the final verification results obtained under the response surface-optimized conditions.

**Figure 3:**
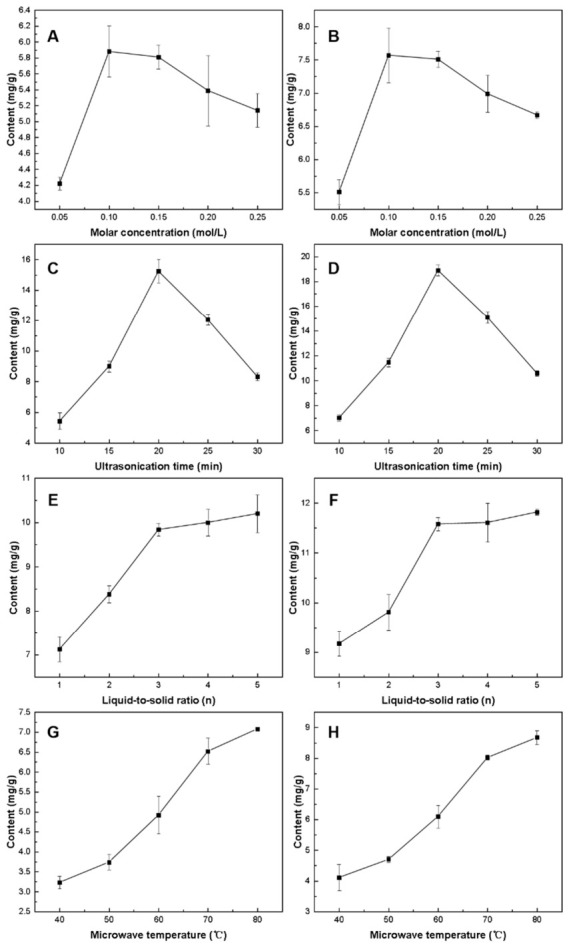
Effects of extraction variables on the recovery of marker monoterpene glycosides from *Radix Paeoniae Alba*. (**A**,**B**) Ionic liquid concentration; (**C**,**D**) ultrasonic time; (**E**,**F**) solid-to-liquid ratio; (**G**,**H**) microwave temperature. Panels (**A**,**C**,**E**,**G**) show paeoniflorin content, whereas panels (**B**,**D**,**F**,**H**) show total marker monoterpene glycoside content. Data are expressed as mean ± SD (n = 3). Contents are expressed as mg/g dry material.

The main variables selected for subsequent response surface optimization are shown in Figure 3. Ionic liquid concentration affected the extraction response within the tested range. When the concentration increased from 0.05 to 0.10 mol/L, the contents of both paeoniflorin and total marker monoterpene glycosides increased. Further increasing the concentration led to a decrease in the response. This trend may be associated with a balance between improved solvent–solute interaction at moderate ionic liquid concentrations and possible mass-transfer limitations at higher concentrations. However, because physicochemical parameters such as viscosity, polarity, conductivity and pH were not independently measured, this explanation should be regarded as a cautious interpretation rather than a quantitative physicochemical mechanism.

Ultrasonic time also influenced the extraction response. The contents of paeoniflorin and total marker monoterpene glycosides increased as the ultrasonic time was extended from 10 to 20 min. Further prolonging the ultrasonic treatment did not markedly improve the response. A moderate ultrasonic treatment may facilitate solvent penetration and matrix disruption, whereas excessive treatment may provide limited additional benefit under the tested conditions.

The solid-to-liquid ratio showed a clear effect on extraction performance. Increasing the solvent volume improved the extraction response within the lower range, probably because a larger solvent amount increased the concentration gradient and improved contact between the plant powder and extraction solvent. However, after a certain range, further increasing the solvent volume produced only a limited increase or even a decrease in the response, indicating that excessive dilution was not beneficial for practical extraction efficiency.

Microwave temperature also affected the extraction response. Increasing the temperature within a suitable range may promote solvent diffusion and mass transfer. However, an excessively high temperature did not further improve the recovery of the marker compounds and may be unfavorable for maintaining a stable extraction environment. Therefore, microwave temperature was not selected as a response surface variable in the final optimization, while ionic liquid concentration, ultrasonic time and solid-to-liquid ratio were selected for Box–Behnken optimization based on their response trends and practical controllability.

All contents are expressed as mg/g dry material. Total marker monoterpene glycosides were calculated as the sum of oxypaeoniflorin, albiflorin and paeoniflorin.

### 2.5. Response Surface Optimization and Model Validation

Based on the single-factor results, a Box–Behnken design was used to optimize the ionic liquid-assisted sequential ultrasound–microwave extraction process. Three independent variables were selected: solid-to-liquid ratio (A), ionic liquid concentration (B) and ultrasonic time (C). The response variables were paeoniflorin content (Y_1_) and total marker monoterpene glycoside content (Y_2_). A total of 17 experimental runs were performed, including five center-point replicates. The complete experimental matrix and ANOVA results are provided in Supplementary Table S3.

Before discussing the fitted models, the response surface design, model summary and verification results are summarized in Table 2. For paeoniflorin, the fitted second-order polynomial equation was:

Y_1_ = 28.69 + 1.76A + 2.53B + 1.35C − 0.2525AB − 0.3525AC + 1.36BC − 3.51A^2^ − 3.91B^2^ − 2.25C^2^

where Y_1_ represents paeoniflorin content, and A, B and C represent solid-to-liquid ratio, ionic liquid concentration and ultrasonic time, respectively. The model was highly significant, with an F-value of 37.81 and *p*-value < 0.0001. The R2, adjusted R2 and predicted R2 values were 0.9798, 0.9539 and 0.7854, respectively. The coefficient of variation was 3.56%, and the adequate precision value was 18.1431. The lack-of-fit was not significant, with a *p*-value of 0.2233. These results indicated that the model fitted the experimental data well within the tested design space.

For total marker monoterpene glycosides, the fitted second-order polynomial equation was:

Y_2_ = 34.26 + 2.20A + 2.88B + 1.55C − 0.47AB − 0.43AC + 1.44BC − 4.01A^2^ − 4.50B^2^ − 2.78C^2^

where Y_2_ represents the summed content of paeoniflorin, oxypaeoniflorin and albiflorin. This model was also highly significant, with an F-value of 49.36 and *p*-value < 0.0001. The R2, adjusted R2 and predicted R2 values were 0.9845, 0.9645 and 0.7979, respectively. The coefficient of variation was 3.02%, and the adequate precision value was 20.9443. The lack-of-fit was not significant, with a *p*-value of 0.0737. These model statistics supported the adequacy of the fitted model for optimization within the investigated experimental domain.

Among the linear terms, ionic liquid concentration showed the largest effect on both response values, followed by solid-to-liquid ratio and ultrasonic time. The linear terms A, B and C were significant for both models. The quadratic terms A2, B2 and C2 were also significant, indicating curved response surfaces and the presence of an optimal region within the tested ranges. Among the interaction terms, BC was significant, suggesting that ionic liquid concentration and ultrasonic time had an interaction effect on the extraction response.

**Figure 4:**
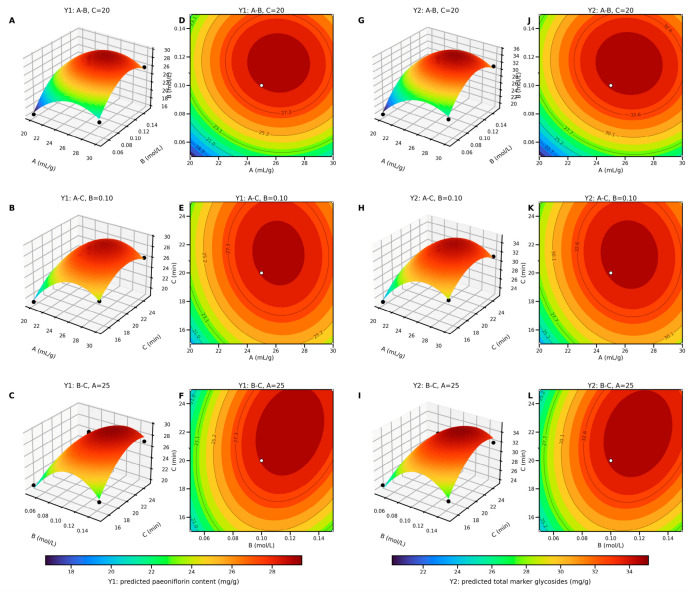
Response surface and contour plots for Box–Behnken optimization of ionic liquid-assisted sequential ultrasound–microwave extraction of marker monoterpene glycosides from Radix Paeoniae Alba. (**A**) Three-dimensional response surface for paeoniflorin content as affected by solid-to-liquid ratio and ionic liquid concentration; (**B**) three-dimensional response surface for paeoniflorin content as affected by solid-to-liquid ratio and ultrasonic time; (**C**) three-dimensional response surface for paeoniflorin content as affected by ionic liquid concentration and ultrasonic time; (**D**) contour plot corresponding to panel (**A**); (**E**) contour plot corresponding to panel (**B**); (**F**) contour plot corresponding to panel (**C**); (**G**) three-dimensional response surface for total marker monoterpene glycoside content as affected by solid-to-liquid ratio and ionic liquid concentration; (**H**) three-dimensional response surface for total marker monoterpene glycoside content as affected by solid-to-liquid ratio and ultrasonic time; (**I**) three-dimensional response surface for total marker monoterpene glycoside content as affected by ionic liquid concentration and ultrasonic time; (**J**) contour plot corresponding to panel (**G**); (**K**) contour plot corresponding to panel (**H**); (**L**) contour plot corresponding to panel (**I**). The variable not shown in each plot was kept at its center level. In the response surface and contour plots, the color gradient from blue/green to yellow/red indicates increasing predicted content. Contents are expressed as mg/g dry material.

The response surface and contour plots are shown in Figure 4. The three-dimensional plots showed curved surfaces, and the corresponding contour plots suggested that the interaction between ionic liquid concentration and ultrasonic time was more pronounced than the other two interaction pairs. These plots were used to visualize the model-predicted response trends and should be interpreted together with the ANOVA results.

The predicted optimal conditions were a solid-to-liquid ratio of 1:25.79 g/mL, ionic liquid concentration of 0.12 mol/L and ultrasonic time of 21.60 min. For practical operation, these values were adjusted to a solid-to-liquid ratio of 1:26 (g/mL), ionic liquid concentration of 0.12 mol/L and ultrasonic time of 22 min. Under these conditions, the experimental paeoniflorin content was 29.12 mg/g dry material, with an RSD of 2.02%, and the total marker monoterpene glycoside content was 34.98 mg/g dry material, with an RSD of 1.02%. The relative errors between predicted and experimental values were 1.62% for paeoniflorin and 0.85% for total marker monoterpene glycosides.

The verification experiment supported the adequacy of the response surface models for locating a practical optimum near the predicted region. However, because validation was performed at the predicted optimum rather than at multiple independent points across the entire design space, the models should be interpreted as optimization tools for the investigated factor ranges rather than as broadly generalizable predictive models.

### 2.6. UPLC-QTOF-MS Profiling of the Optimized Extract

To obtain a broader chemical profile of the optimized ionic liquid-assisted sequential ultrasound–microwave extract of *Radix Paeoniae Alba*, UPLC-QTOF-MS analysis was performed in both positive and negative electrospray ionization modes. This analysis was used as a non-targeted profiling approach to complement the targeted HPLC quantification of oxypaeoniflorin, albiflorin and paeoniflorin.

The base peak ion chromatograms of the optimized extract in positive and negative ion modes are provided in Figure S3 as supplementary chromatographic overview information. These chromatograms showed multiple detectable peaks over the chromatographic gradient, indicating the chemical complexity of the optimized extract. However, global chromatographic profiles should not be interpreted as definitive structural identification evidence.

Representative candidate features are summarized in Table 3, and a fuller list is provided in Table S4. Candidate features were screened and annotated mainly based on accurate mass, ion mode, retention behavior, isotope pattern, adduct information and database matching. Because authentic standards were not available for all non-targeted candidates and representative raw MS/MS spectra were not available for reliable fragment-ion or neutral-loss interpretation, these compounds are reported as database-assisted tentative annotations rather than definitive identifications.

Several Paeonia-related monoterpene glycoside candidates were tentatively annotated in the optimized extract, including oxypaeoniflorin-related, paeoniflorin-related and benzoylpaeoniflorin-related features. Galloylated glucose derivatives and polyphenolic candidates were also observed, suggesting that the optimized extract contained multiple polar constituent classes.

Nevertheless, because a matched UPLC-QTOF-MS comparison with the conventional reflux extract was not performed, the present profiling results describe the chemical features of the optimized extract but do not demonstrate selective enhancement or compositional differences relative to conventional extraction. Unambiguous structural identification of non-targeted constituents would require authentic standards, detailed MS/MS interpretation and, when necessary, additional spectroscopic evidence.

### 2.7. Molecular Interaction Evidence and Proposed Extraction-Enhancement Mechanism

To provide molecular-level insight into the possible role of the selected ionic liquid, paeoniflorin was used as a representative monoterpene glycoside for computational and NMR analyses. Paeoniflorin contains multiple hydroxyl, ether and ester groups, which may provide potential interaction sites with both the imidazolium cation and the dihydrogen phosphate anion of 1-propyl-3-methylimidazolium dihydrogen phosphate. Because the calculations were performed using simplified molecular systems, the results should be interpreted as supportive molecular interaction evidence rather than a complete representation of the real plant-matrix extraction environment.

The electrostatic potential surface of paeoniflorin showed distinct positive and negative electrostatic regions. Positive electrostatic regions were mainly distributed around hydroxyl hydrogens, whereas negative electrostatic regions were associated with oxygen-containing groups. This uneven electrostatic distribution suggested that paeoniflorin has potential sites for electrostatic and hydrogen bonding-related interactions with ionic liquid components. However, the electrostatic potential map alone does not prove the formation of specific interactions during extraction.

SAPT energy decomposition was used to evaluate the interaction components in the simplified paeoniflorin-[C3MIM][H_2_PO_4_] binary complex. In this simplified model, electrostatic interaction showed the largest attractive contribution, while induction and dispersion interactions also contributed to complex stabilization. Therefore, electrostatic interaction appeared to be an important contributor in the calculated binary model. This result should not be interpreted as definitive proof that electrostatic interaction is the dominant factor in the complete plant-matrix extraction system.

Non-covalent interaction analysis provided a qualitative visualization of possible interaction regions between paeoniflorin and the selected ionic liquid. Hydrogen bonding-related regions were observed near oxygen-containing groups, and van der Waals interaction regions were also present around molecular contact areas. These results were consistent with the possibility that multiple non-covalent interactions may participate in paeoniflorin–ionic liquid association. Nevertheless, the NCI results were derived from simplified optimized structures and cannot fully reproduce solvent effects, plant-matrix effects or the conformational flexibility of paeoniflorin in the real extraction system.

^1^H NMR spectra of paeoniflorin before and after ionic liquid addition were compared to provide experimental support for possible intermolecular interactions. Changes in the 3.0–4.2 ppm region and peak shape/chemical shift perturbations were observed after ionic liquid addition, suggesting changes in the local electronic environment of paeoniflorin-related proton signals. This chemical shift perturbation was consistent with possible paeoniflorin–ionic liquid interactions. However, because complete signal assignment, two-dimensional NMR confirmation and concentration-dependent NMR analysis were not performed, the present NMR evidence should not be regarded as definitive confirmation of a specific paeoniflorin–ionic liquid complex.

Based on these computational and NMR results, a process-level interpretation of ionic liquid-assisted sequential ultrasound–microwave extraction was proposed. At the process level, ultrasound treatment may facilitate matrix disruption, solvent penetration and mass transfer, whereas microwave heating may promote solvent heating and molecular diffusion. At the molecular level, the selected ionic liquid may interact with paeoniflorin and structurally related monoterpene glycosides through hydrogen bonding-related, electrostatic and dispersion interactions, thereby contributing to solubilization and migration of the marker glycosides into the extraction solvent.

**Figure 5:**
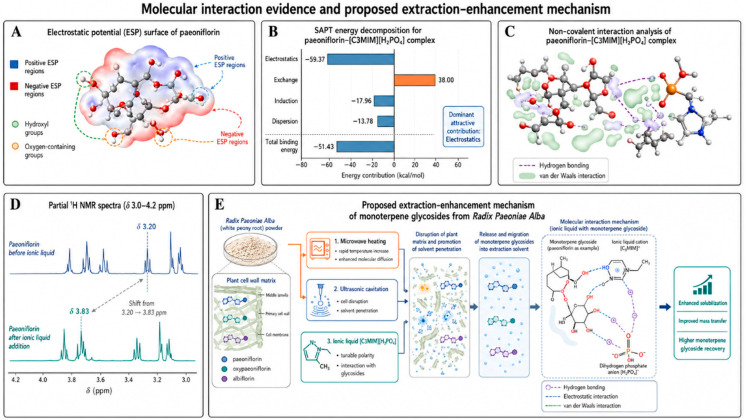
Molecular interaction evidence and proposed process-level interpretation of ionic liquid-assisted sequential ultrasound–microwave extraction. (**A**) Electrostatic potential surface of paeoniflorin. (**B**) SAPT energy decomposition of the simplified paeoniflorin-[C3MIM][H_2_PO_4_] binary complex. (**C**) Non-covalent interaction visualization of the simplified paeoniflorin-[C3MIM][H_2_PO_4_] binary model. (**D**) Partial ^1^H NMR spectra of paeoniflorin before and after ionic liquid addition, showing representative chemical shift perturbation. (**E**) Proposed process-level interpretation involving ultrasound-assisted matrix disruption, microwave-assisted heating and diffusion, and ionic liquid-assisted solubilization of representative monoterpene glycosides. The computational panels represent simplified molecular models and should not be interpreted as complete representations of the real plant-matrix extraction environment. Additional ESP views, SAPT results, NCI plots and full ^1^H NMR spectra are provided in Supplementary Figures S4–S7. AI assistance was used only for schematic layout preparation; all scientific data shown in the figure were derived from the authors’ original results.

Overall, the ESP, SAPT, NCI and ^1^H NMR results provide supportive evidence for possible molecular interactions between paeoniflorin and the selected ionic liquid. These results support a plausible molecular interaction explanation for the improved extraction response, but they do not conclusively establish the complete extraction mechanism in the real plant matrix. Further studies involving broader conformational sampling, explicit solvent models, plant-matrix simulations, two-dimensional NMR analysis and additional spectroscopic evidence would be needed to fully clarify the detailed interaction mechanism (Figure 5).

## 3. Materials and Methods

### 3.1. Plant Material and Reagents

Dried roots of *Paeonia lactiflora* Pall., namely, *Radix Paeoniae Alba*, were purchased from Songyaotang medicinal materials supplier, Bozhou, Anhui Province, China. The plant material was pulverized using a JC-FW-400A grinder (Qingdao Juchuang Environmental Protection Group Co., Ltd., Qingdao, China) and passed through a 45-mesh sieve before extraction experiments.

Paeoniflorin, oxypaeoniflorin and albiflorin reference standards were purchased from Beijing Solarbio Science & Technology Co., Ltd. (Beijing, China), with HPLC purity ≥98%. Acetonitrile and methanol were of chromatographic grade. Phosphoric acid and ethanol were of analytical grade. An ethanol–water solution was used as the extraction solvent. The ionic liquids used for screening included imidazolium-based ionic liquids with different anions and alkyl chain lengths. The selected ionic liquid for subsequent experiments was 1-propyl-3-methylimidazolium dihydrogen phosphate. The detailed ionic liquid codes, names, suppliers and purities are provided in Supplementary Table S2. All other reagents were of analytical grade unless otherwise stated.

### 3.2. HPLC Analysis of Paeoniflorin, Oxypaeoniflorin and Albiflorin

The contents of oxypaeoniflorin, albiflorin and paeoniflorin were determined using a Waters e2695 HPLC system equipped with a UV detector (Waters, Milford, MA, USA). Separation was performed on an InertSustain C18 column (4.6 × 250 mm, 5 μm). The mobile phase consisted of acetonitrile (A) and 0.05% phosphoric acid aqueous solution (B). The gradient elution program was as follows: 0–15 min, 14% A; 15–16 min, 14–17% A; 16–20 min, 17% A; 20–25 min, 14% A. The flow rate was 1.0 mL/min, and the injection volume was 10 µL. Oxypaeoniflorin was detected at 258 nm, whereas albiflorin and paeoniflorin were detected at 230 nm.

Stock solutions of paeoniflorin, oxypaeoniflorin and albiflorin were prepared in methanol. Briefly, 5.10 mg of paeoniflorin, 5.02 mg of oxypaeoniflorin and 5.17 mg of albiflorin were accurately weighed, dissolved in methanol and diluted to volume. Equal volumes of the three standard solutions were mixed to obtain a mixed standard stock solution containing paeoniflorin, oxypaeoniflorin and albiflorin at concentrations of 0.255, 0.251 and 0.259 mg/mL, respectively.

For sample preparation, the extract was dissolved or diluted with methanol to an appropriate volume and filtered through a 0.45 μm membrane before HPLC injection. Calibration curves were constructed by injecting different amounts of the mixed standard solution. The HPLC method was evaluated in terms of selectivity, linearity, precision, repeatability, sensitivity and recovery-based trueness. LOD and LOQ were estimated using signal-to-noise ratios of approximately 3 and 10, respectively. Recovery-based trueness was evaluated by spiking known amounts of mixed standards into sample solutions. The total marker monoterpene glycoside content was calculated as the sum of oxypaeoniflorin, albiflorin and paeoniflorin and expressed as mg/g dry material.

### 3.3. Conventional Reflux Extraction and Sequential Ultrasound–Microwave-Assisted Extraction

To compare the extraction efficiency of conventional and assisted extraction methods, conventional reflux extraction and sequential ultrasound–microwave-assisted extraction were performed. For conventional reflux extraction, powdered *Radix Paeoniae Alba* was extracted using a 70% ethanol–water solution (*v*/*v*) under reflux conditions. The reflux extraction time was varied according to the experimental design. After extraction, the mixture was cooled, treated to remove insoluble residues, diluted to an appropriate volume, filtered through a 0.45 μm membrane and analyzed by HPLC.

For sequential ultrasound–microwave-assisted extraction, powdered *Radix Paeoniae Alba* was first treated using a KH-500DE ultrasonic cleaner (Kunshan Hechuang Co., Ltd., Kunshan, China) and then subjected to microwave-assisted extraction using an XH-100A microwave catalytic synthesis/extraction instrument (Beijing Xianghu Technology Co., Ltd., Beijing, China). In the preliminary comparison experiment, ultrasound treatment was performed for 20 min, followed by microwave treatment for 10 min. The resulting extract was centrifuged using a DT5-4B low-speed centrifuge (Beijing Shidai Beili Co., Ltd., Beijing, China) or allowed to stand until insoluble residues were removed. The supernatant was collected, diluted when necessary, filtered through a 0.45 μm membrane and analyzed by HPLC. The extraction efficiency was evaluated using paeoniflorin content and total marker monoterpene glycoside content as response values.

### 3.4. Ionic Liquid Screening Procedure

Representative imidazolium-based ionic liquids with different anions and alkyl chain lengths were selected for comparative screening. The screening was designed as an exploratory structure–extraction response comparison. The ionic liquids used in the screening experiments are listed in Supplementary Table S2.

For anion screening, ionic liquids with the same imidazolium cation but different anions were compared. The tested ionic liquids were coded as L1–L5. The extraction system without ionic liquid was used as the blank control. The extraction conditions were as follows: 70% ethanol–water solution as the extraction solvent, ionic liquid concentration of 0.10 mol/L, solid-to-liquid ratio of 1:20 g/mL, ultrasonic power of 100 W and ultrasonic time of 60 min. After extraction, the solution was allowed to stand until insoluble residues settled, and the supernatant was collected for HPLC analysis.

After selecting the dihydrogen phosphate anion, imidazolium dihydrogen phosphate ionic liquids with different alkyl chain lengths were further compared. The tested ionic liquids were coded as T1–T5. The same extraction and HPLC analysis procedures were used. Paeoniflorin content and total marker monoterpene glycoside content were used as response values. All screening experiments were performed in triplicate, and the results are expressed as mean ± SD.

### 3.5. Single-Factor Experiments

Single-factor experiments were performed after ionic liquid screening to evaluate the effects of major extraction parameters on paeoniflorin content and total marker monoterpene glycoside content. The selected ionic liquid, 1-propyl-3-methylimidazolium dihydrogen phosphate, was used in these experiments.

Unless otherwise specified, the initial extraction conditions were as follows: solid-to-liquid ratio of 1:20 g/mL, 70% ethanol–water solution containing the selected ionic liquid, ultrasonic time of 10 min, ultrasonic power of 300 W, microwave power of 300 W, microwave temperature of 45 °C and microwave time of 10 min. For each single-factor experiment, only one variable was changed, while the other conditions were kept constant.

The investigated variables included ionic liquid concentration, ultrasonic time, ultrasonic power, solid-to-liquid ratio and microwave temperature. The tested ionic liquid concentrations were 0.05, 0.10, 0.15, 0.20 and 0.25 mol/L. The tested ultrasonic times were 10, 15, 20, 25 and 30 min. The tested ultrasonic powers were 100, 150, 200, 250 and 300 W. The tested solid-to-liquid ratios were 1:15, 1:20, 1:25, 1:30 and 1:35 g/mL. The tested microwave temperatures were 40, 50, 60, 70 and 80 °C. After extraction, the samples were centrifuged or allowed to stand until insoluble residues were removed. The supernatants were collected, diluted when necessary, filtered through a 0.45 μm membrane and analyzed by HPLC. All single-factor experiments were performed in triplicate, and the results are expressed as mean ± SD.

### 3.6. Response Surface Methodology

Based on the single-factor experiments, three major factors were selected for response surface optimization: solid-to-liquid ratio, ionic liquid concentration and ultrasonic time. A Box–Behnken design with three factors and three levels was used to optimize the extraction process. The independent variables were coded as follows: A, solid-to-liquid ratio; B, ionic liquid concentration; and C, ultrasonic time. The levels of the three factors were set as follows: solid-to-liquid ratio, 1:20, 1:25 and 1:30 g/mL; ionic liquid concentration, 0.05, 0.10 and 0.15 mol/L; ultrasonic time, 15, 20 and 25 min.

The response variables were paeoniflorin content and total marker monoterpene glycoside content. A total of 17 experimental runs were performed according to the Box–Behnken design, including five center-point replicates. The experimental data were fitted to a second-order polynomial model:

Y = β_0_ + Σβ_i_X_i_ + Σβ_i__i_X_i_^2^ + Σβ_i__j_X_i_X_j_

where Y is the predicted response; beta0 is the intercept coefficient; beta_i, beta_ii and beta_ij are the linear, quadratic and interaction coefficients, respectively; and X_i and X_j represent the coded independent variables.

Analysis of variance was used to evaluate the significance and adequacy of the fitted models. Model adequacy was assessed using model F-value, *p*-value, R2, adjusted R2, predicted R2, coefficient of variation, lack-of-fit test and adequate precision. The optimal extraction conditions predicted by the model were verified experimentally in triplicate. The relative error between predicted and experimental values was calculated to evaluate the agreement between predicted and experimental values near the optimum.

### 3.7. UPLC-QTOF-MS Analysis

UPLC-QTOF-MS analysis was performed using a Waters G2-XS QTOF mass spectrometer coupled with a Waters H-Class UPLC system (Milford, MA, USA). UNIFI software (version 1.9.3) was used for data processing. For sample preparation, approximately 3 mg of sample was treated with the extraction solvent according to the analytical protocol, vortexed for 10 min and centrifuged at 12,000 r/min for 10 min. The supernatant was collected for UPLC-QTOF-MS analysis.

Chromatographic separation was performed on a Waters BEH C18 column (1.7 μm, 2.1 × 50 mm). The mobile phase consisted of 0.1% formic acid in water (A) and 0.1% formic acid in acetonitrile (B). The column temperature was maintained at 40 °C, the flow rate was 0.4 mL/min and the injection volume was 2 μL. The gradient program was as follows: 0–1 min, 5% B; 1–35 min, 5–98% B; 35–40 min, 98% B.

Mass spectrometric detection was performed using an electrospray ionization source in both positive and negative ion modes. Data were acquired in MSe profile mode over an m/z range of 50–1200. Argon was used as the collision gas. The nebulizing gas flow rate was 800 L/h, the cone gas flow rate was 50 L/h, the desolvation temperature was 400 °C, the source temperature was 110 °C, and the capillary voltage was 2.0 kV in both ion modes. The collision energy was set at 20–40 V, and sodium formate was used for mass-axis calibration.

For database-assisted screening, acquired data were processed using UNIFI software. Compound structural files related to *Radix Paeoniae Alba* constituents were obtained from ChemSpider or PubChem and imported into UNIFI to establish an in-house screening database containing molecular structures and exact molecular masses. The selected adduct ions included [M + H]^+^, [M + Na]^+^ and [M − H]^−^. The mass tolerance for precursor and fragment-related matching was set at 10 ppm, and isotope patterns were considered during candidate screening. Because representative raw MS/MS spectra were not available for all candidate features, the UPLC-QTOF-MS results were reported as database-assisted tentative annotations.

### 3.8. Computational Analysis

The initial structures of paeoniflorin and the selected ionic liquid were constructed and pre-optimized using Avogadro (version 1.99.0, Avogadro2) with the MMFF94 force field. The structures were further pre-optimized using the GFN2-xTB method. Possible paeoniflorin–ionic liquid complexes were searched using ABCluster (version 1.4) to obtain selected favorable conformations. Further geometry optimization was performed using ORCA (version 5.0.4) at the PBEh-3c/def2-SVP level. The calculations were performed on selected optimized molecular models and were used to provide qualitative molecular interaction insight rather than exhaustive conformational analysis.

Wavefunction files were generated from the optimized structures for subsequent surface and interaction analyses. Electrostatic potential surface analysis and non-covalent interaction analysis were performed using Multiwfn (version 3.7), and the corresponding molecular surfaces and interaction regions were visualized using VMD (version 1.9.3). SAPT energy decomposition was performed using Psi4 (version 1.8) at the SAPT0/jun-cc-pVDZ level to estimate interaction energy components in the simplified paeoniflorin–ionic liquid binary model. The computational results were interpreted as supportive molecular interaction evidence based on simplified models.

### 3.9. ^1^H NMR Analysis

^1^H NMR analysis was performed to provide experimental support for possible interaction between paeoniflorin and the selected ionic liquid. Paeoniflorin solution and paeoniflorin solution containing the selected ionic liquid were prepared in DMSO-*d*_6_ and analyzed under the same instrumental conditions. The spectra were acquired using a Bruker 400 MHz spectrometer at 297.3 K with a 5 mm PABBO BB/19F-1H/D Z-GRD probe (Billerica, MA, USA). A one-dimensional ^1^H NMR experiment was performed using the zg30 pulse sequence. The number of scans was 8, the receiver gain was 56.5, the relaxation delay was 1.0000 s, the pulse width was 10.0000 µs, the acquisition time was 2.0447 s, and the spectrometer frequency was 400.13 MHz.

The spectra were compared to evaluate chemical shift perturbation and peak shape changes after ionic liquid addition. Chemical shift changes were used as supportive evidence for changes in the local electronic environment of paeoniflorin in the presence of the ionic liquid. Because complete signal assignment, two-dimensional NMR confirmation and concentration-dependent NMR analysis were not performed, the NMR results were interpreted cautiously and were not used as definitive proof of a specific paeoniflorin–ionic liquid complex.

### 3.10. Statistical Analysis

All extraction experiments were performed in triplicate unless otherwise specified. Results are expressed as mean ± SD when replicate data are available. Response surface methodology and analysis of variance were performed using Design-Expert 13.0 software. For RSM models, *p*-values were used to evaluate the significance of model terms, and lack-of-fit tests were used to assess model adequacy. Significance markers were not added to figures unless supported by formal statistical analysis. The relative error between predicted and experimental values in the model verification experiment was calculated using the following equation:

Relative error (%) = |Predicted value − Experimental value|/Predicted value × 100%

## 4. Conclusions

In this study, an ionic liquid-assisted sequential ultrasound–microwave extraction process was developed for the recovery of representative monoterpene glycosides from *Radix Paeoniae Alba*. Paeoniflorin, oxypaeoniflorin and albiflorin were selected as marker compounds, and their summed content was used as the total marker monoterpene glycoside index. Among the tested ionic liquids, 1-propyl-3-methylimidazolium dihydrogen phosphate showed the highest extraction response and was selected for subsequent optimization. Response surface methodology gave practical extraction conditions of a solid-to-liquid ratio of 1:26 g/mL, ionic liquid concentration of 0.12 mol/L and ultrasonic time of 22 min. Under these conditions, paeoniflorin and total marker monoterpene glycoside contents reached 29.12 and 34.98 mg/g dry material, respectively.

UPLC-QTOF-MS profiling provided a broader chemical view of the optimized extract and enabled database-assisted tentative annotation of representative Paeonia-related candidate features, including monoterpene glycoside derivatives, galloylated glucose derivatives and polyphenolic candidates. These annotations should be interpreted according to their evidence levels, and compounds without authentic-standard confirmation and reliable MS/MS interpretation should be regarded as tentative annotations rather than definitive identifications. Because a matched UPLC-QTOF-MS comparison with conventional reflux extract was not performed, the present profiling results describe the chemical features of the optimized extract but do not demonstrate selective enhancement or compositional differences relative to conventional extraction.

Computational analysis and ^1^H NMR chemical shift perturbation provided supportive molecular-level information for possible interactions between paeoniflorin and the selected ionic liquid. In the simplified paeoniflorin–ionic liquid binary model, electrostatic interaction appeared to be an important contributor, while induction, dispersion and hydrogen bonding-related interactions may also participate. Together, the computational and NMR results indicate that ionic liquid–paeoniflorin interactions may contribute to the improved extraction response, while the complete mechanism in the plant matrix requires further verification.

Overall, the developed method provides an optimized multi-marker extraction approach for *Radix Paeoniae Alba* and offers cautious molecular interaction insights into ionic liquid-assisted recovery of polar monoterpene glycosides. Future work should further investigate ionic liquid residue control, solvent recovery, process scalability, direct comparisons with ultrasound-only and microwave-only extraction, matched chemical profiling of conventional and optimized extracts, and more comprehensive structural confirmation of non-targeted candidate constituents.

## Figures and Tables

**Table 1:** Selected validation parameters of the HPLC method for three marker monoterpene glycosides.

Analyte	RT (min)	Resolution	Theoretical Plates	Tailing Factor	Linear Range (μg Injected)	r	Mean Recovery (%)
Oxypaeoniflorin	6.351	1.599	6575	1.121	0.0391–2.5	0.9991	101.61
Albiflorin	12.135	13.556	8598	1.066	0.0391–2.5	0.9998	101.00
Paeoniflorin	14.995	4.903	9317	1.100	0.0391–2.5	0.9995	101.49

Note: Detailed calibration equations, LOD/LOQ, precision and repeatability data are provided in Table S1.

**Table 2:** Box–Behnken design factors, model statistics and verification results for ionic liquid-assisted sequential ultrasound–microwave extraction.

Item	Variable/Response	Level or Value
Factor A	Solid-to-liquid ratio (g/mL)	1:20, 1:25, 1:30
Factor B	Ionic liquid concentration (mol/L)	0.05, 0.10, 0.15
Factor C	Ultrasonic time (min)	15, 20, 25
Response Y_1_	Paeoniflorin content (mg/g)	Model fitted by BBD
Response Y_2_	Total marker monoterpene glycoside content (mg/g)	Model fitted by BBD
Model F-value for Y_1_	Paeoniflorin model	37.81
Model *p*-value for Y_1_	Paeoniflorin model	<0.0001
R^2^ for Y_1_	Paeoniflorin model	0.9798
Lack-of-fit *p*-value for Y_1_	Paeoniflorin model	0.2233
Adequate precision for Y_1_	Paeoniflorin model	18.1431
Model F-value for Y_2_	Total marker monoterpene glycoside model	49.36
Model *p*-value for Y_2_	Total marker monoterpene glycoside model	<0.0001
R^2^ for Y_2_	Total marker monoterpene glycoside model	0.9845
Lack-of-fit *p*-value for Y_2_	Total marker monoterpene glycoside model	0.0737
Adequate precision for Y_2_	Total marker monoterpene glycoside model	20.9443
Predicted optimal condition	A, B, C	1:25.79 g/mL, 0.12 mol/L, 21.60 min
Practical optimal condition	A, B, C	1:26 g/mL, 0.12 mol/L, 22 min
Predicted Y_1_	Paeoniflorin content	29.60 mg/g
Experimental Y_1_	Paeoniflorin content	29.12 mg/g, RSD 2.02%
Relative error for Y_1_	Paeoniflorin content	1.62%
Predicted Y_2_	Total marker monoterpene glycoside model	35.28 mg/g
Experimental Y_2_	Total marker monoterpene glycoside model	34.98 mg/g, RSD 1.02%
Relative error for Y_2_	Total marker monoterpene glycoside model	0.85%

Note: Adjusted R^2^ values for Y_1_/Y_2_ were 0.9539/0.9645; predicted R^2^ values for Y_1_/Y_2_ were 0.7854/0.7979; and CV values for Y_1_/Y_2_ were 3.56%/3.02%, respectively.

**Table 3:** Representative database-assisted tentative candidate features detected in the optimized extract by UPLC-QTOF-MS.

No.	Class	Putative Assignment	Formula	Ion Mode	RT (min)	Observed m/z	Observed ion	Mass Error (ppm)	Evidence
1	Monoterpene glycoside	Oxypaeoniflorin	C_23_H_28_O_12_	ESI−	1.74	495.1497	[M − H]^−^	−2.1	Tentative; accurate mass + database match
2	Monoterpene glycoside derivative	Benzoylpaeoniflorin	C_30_H_32_O_12_	ESI+	9.39	585.1965	[M + H]^+^	−0.2	Tentative; accurate mass + database match
3	Galloylated monoterpene glycoside	Galloylpaeoniflorin	C_30_H_32_O_15_	ESI−	5.29	631.1673	[M − H]^−^	0.8	Tentative; accurate mass + database match
4	Galloylated monoterpene glycoside	Galloyloxypaeoniflorin	C_30_H_32_O_16_	ESI−	5.46	647.1600	[M − H]^−^	−2.7	Tentative; accurate mass + database match
5	Monoterpene glycoside derivative	Oxidized benzoylpaeoniflorin	C_30_H_32_O_13_	ESI−	9.21	599.1753	[M − H]^−^	−2.9	Tentative; accurate mass + database match
6	Phenolic acid	Gallic acid	C_7_H_6_O_5_	ESI−	0.65	169.0143	[M − H]^−^	0.4	Tentative; accurate mass + database match
7	Galloylated sugar	Galloylglucose	C_13_H_16_O_10_	ESI−	0.57	331.0672	[M − H]^−^	0.5	Tentative; accurate mass + database match
8	Gallotannin	1,6-Digalloyl-β-D-glucose	C_20_H_20_O_14_	ESI−	2.27	483.0773	[M − H]^−^	−1.5	Tentative; accurate mass + database match
9	Gallotannin	1,2,6-Trigalloyl-β-D-glucose	C_27_H_24_O_18_	ESI−	3.50	635.0873	[M − H]^−^	−2.7	Tentative; accurate mass + database match
10	Gallotannin	1,2,3,4,6-Pentagalloyl-β-D-glucose	C_41_H_32_O_26_	ESI−	5.62	939.1115	[M − H]^−^	0.7	Tentative; accurate mass + database match

Note: Candidate features were annotated mainly based on accurate mass, ion mode, retention behavior, isotope pattern, adduct information and database matching. Authentic-standard confirmation and representative raw MS/MS fragment or neutral-loss evidence were not available for all non-targeted candidates; therefore, these features are reported as tentative annotations rather than definitive structural identifications.

## Data Availability

The data presented in this study are available in the article and the Supplementary Materials. Additional raw data are available from the corresponding author upon reasonable request.

## Supplementary Materials

The following supporting information can be downloaded at: https://www.mdpi.com/article/10.3390/molecules31132342/s1, Figure S1: Mixed standard chromatograms and additional HPLC chromatograms for selectivity evaluation using a non-target matrix and spiked mixed standards; Figure S2: Effect of ultrasonic power on the extraction of marker monoterpene glycosides from *Radix Paeoniae Alba*; Figure S3: UPLC-QTOF-MS base peak ion chromatograms of the optimized extract in positive and negative ion modes; Figure S4: Additional electrostatic potential surface views of paeoniflorin; Figure S5: Additional SAPT energy decomposition results and selected optimized molecular models; Figure S6: Additional non-covalent interaction plots of the simplified paeoniflorin–ionic liquid model; Figure S7: Full ^1^H NMR spectra of paeoniflorin before and after ionic liquid addition; Table S1: Detailed HPLC method validation data for oxypaeoniflorin, albiflorin and paeoniflorin; Table S2: Ionic liquids used for anion and alkyl-chain-length screening; Table S3: Complete Box–Behnken design matrix and ANOVA results for response surface models; Table S4: Representative UPLC-QTOF-MS database-assisted candidate features in the optimized extract.
