# Supplementary material for: Ionic Liquid-Assisted Sequential Ultrasound–Microwave Extraction of Monoterpene Glycosides from Radix Paeoniae Alba: Multi-Marker Optimization, UPLC-QTOF-MS Profiling and Molecular Interaction Insights
Source: Molecules. 2026 Jul 3;31(13):2342. doi: 10.3390/molecules31132342 (PMC13363496; doi:10.3390/molecules31132342)
Supplement: Supplementary file 1 [file molecules-31-02342-s001.zip › molecules-4351683-supplementary.pdf]

Supporting Information

Ionic Liquid-Assisted Sequential Ultrasound–Microwave Extraction of Monoterpene Glycosides from *Radix Paeoniae Alba*: Multi-Marker Optimization, UPLC-QTOF-MS Profiling and Molecular Interaction Insights

Jiachen Shen, Jieru Zhang, Xiaoming Peng*, Ying Yang

* Correspondence: pengxiaoming@bipt.edu.cn

Contents

• Figure S1. Mixed standard chromatograms and additional HPLC chromatograms for selectivity evaluation using a non-target matrix and spiked mixed standards.
• Figure S2. Effect of ultrasonic power on the extraction of marker monoterpene glycosides from *Radix Paeoniae Alba*.
• Figure S3. UPLC-QTOF-MS base peak ion chromatograms of the optimized extract in positive and negative ion modes.
• Figure S4. Additional electrostatic potential surface views of paeoniflorin.
• Figure S5. Additional SAPT energy decomposition results and selected optimized molecular models.
• Figure S6. Additional non-covalent interaction plots of the simplified paeoniflorin–ionic liquid model.
• Figure S7. Full ^{1}H NMR spectra of paeoniflorin before and after ionic liquid addition.
• Table S1. Detailed HPLC method validation data for oxypaeoniflorin, albiflorin and paeoniflorin.
• Table S2. Ionic liquids used for anion and alkyl-chain-length screening.
• Table S3. Complete Box–Behnken design matrix and ANOVA results for response surface models.
• Table S4. Representative UPLC-QTOF-MS database-assisted candidate features in the optimized extract.

Evidence boundary note: UPLC-QTOF-MS candidate features were reported as database-assisted tentative annotations. No MS/MS-based neutral-loss table was included because representative raw MS/MS spectra were not available for reliable reporting.

Supplementary Figures

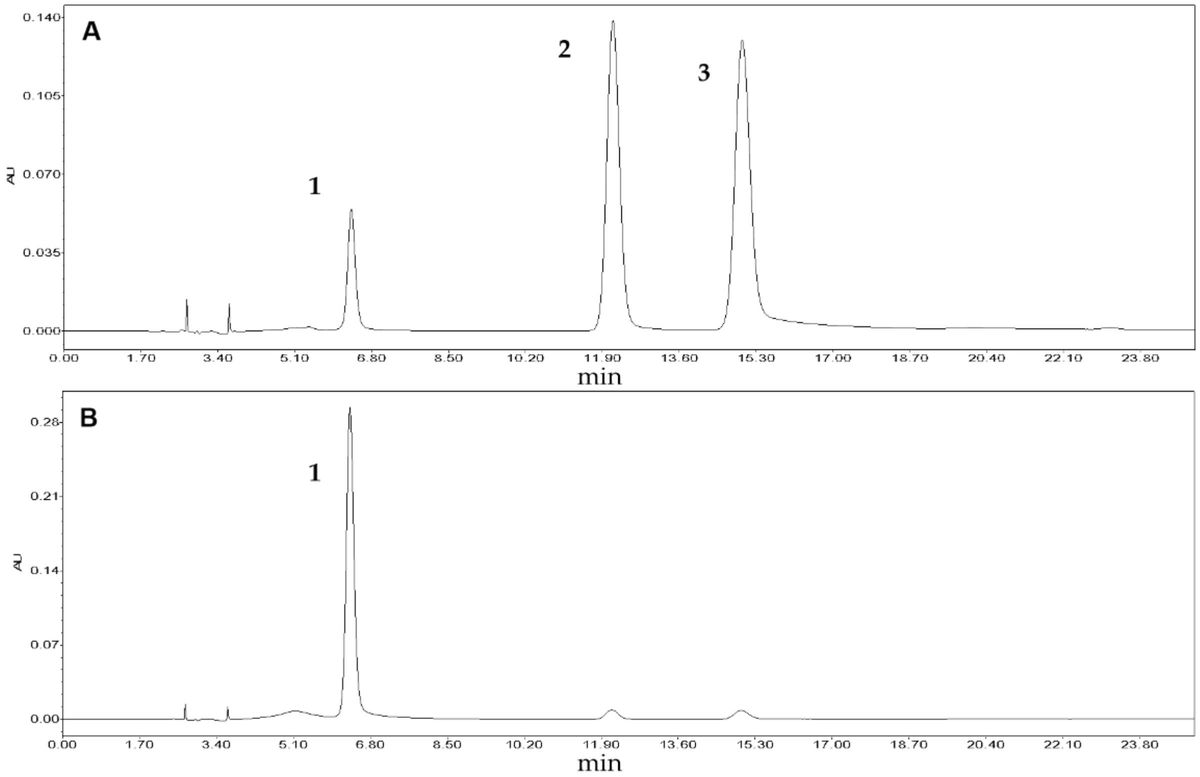
A
B
1
2
3
AU
min
0.140
0.105
0.070
0.035
0.000
0.28
0.21
0.14
0.07
0.00
0.00
1.70
3.40
5.10
6.80
8.50
10.20
11.90
13.60
15.30
17.00
18.70
20.40
22.10
23.80

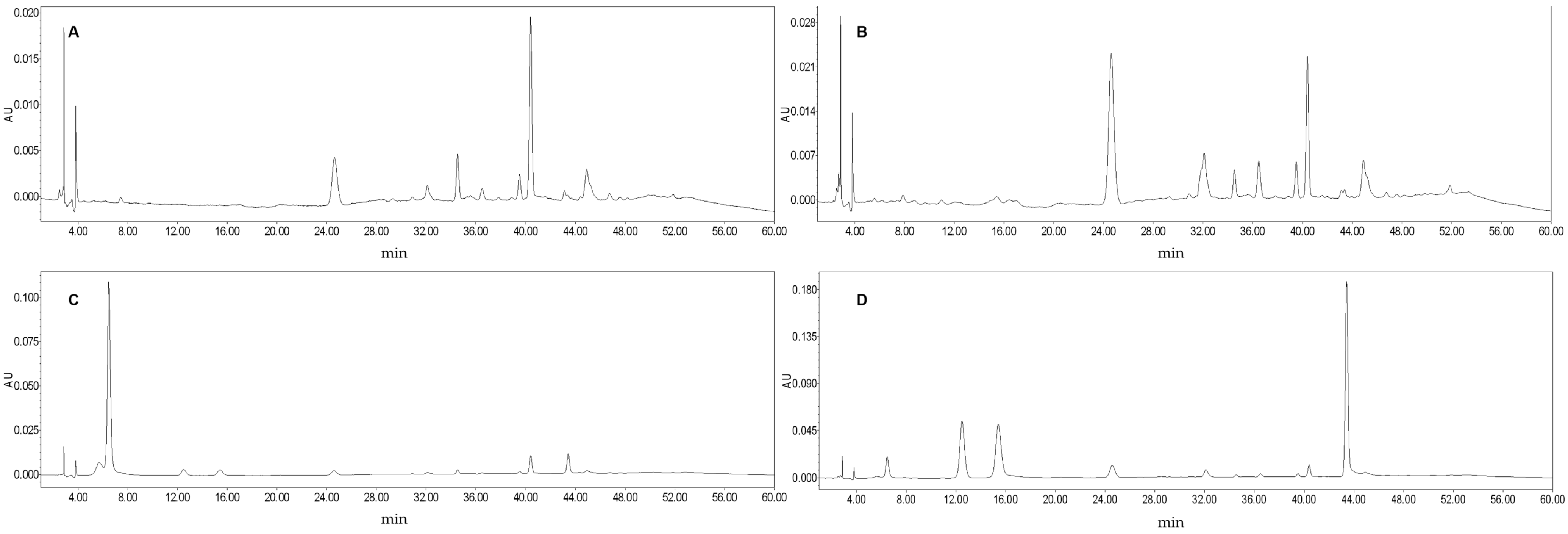

Figure S1. Mixed standard chromatograms and additional HPLC chromatograms for selectivity evaluation. The first image shows mixed standard chromatograms detected at 230 and 258 nm. The second image shows non-target matrix chromatograms and spiked non-target matrix chromatograms detected at 258 and 230 nm. No obvious peak shoulder, peak splitting or retention-time mismatch was observed at the retention times corresponding to oxypaeoniflorin, albiflorin and paeoniflorin after spiking, supporting the selectivity of the established HPLC method for the present comparative extraction study.

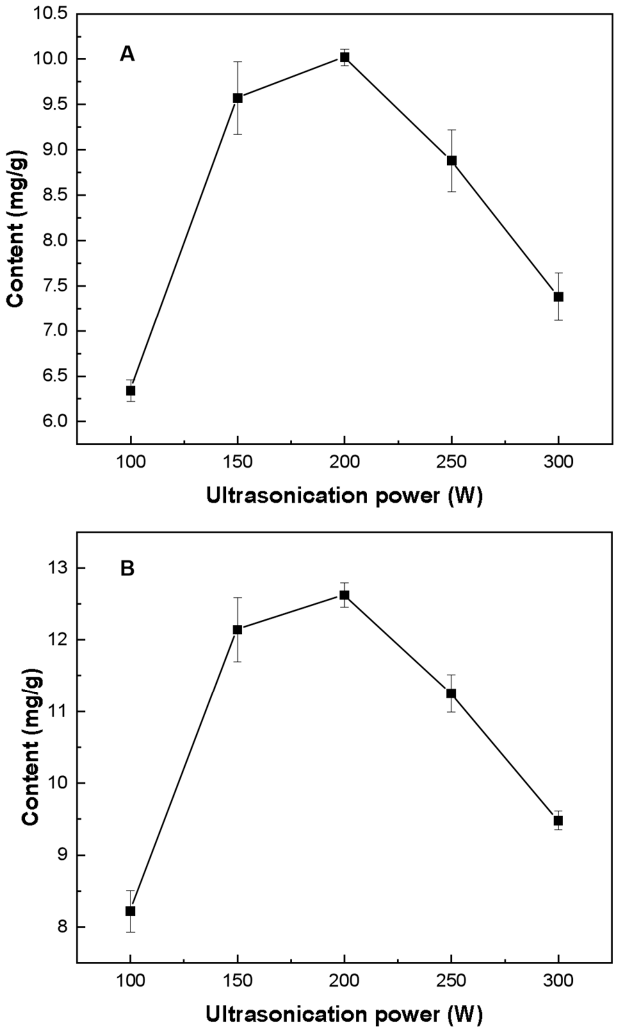

Figure S2. Effect of ultrasonic power on the extraction of marker monoterpene glycosides from *Radix Paeoniae Alba*. (A) Paeoniflorin content; (B) total marker monoterpene glycoside content. Total marker monoterpene glycosides were calculated as the sum of oxypaeoniflorin, albiflorin and paeoniflorin. Because ultrasonic power was not selected as a response surface variable, the corresponding single-factor trend is provided as supplementary information. Contents are expressed as mg/g dry material, and no statistical comparison is made from this supplementary trend plot.

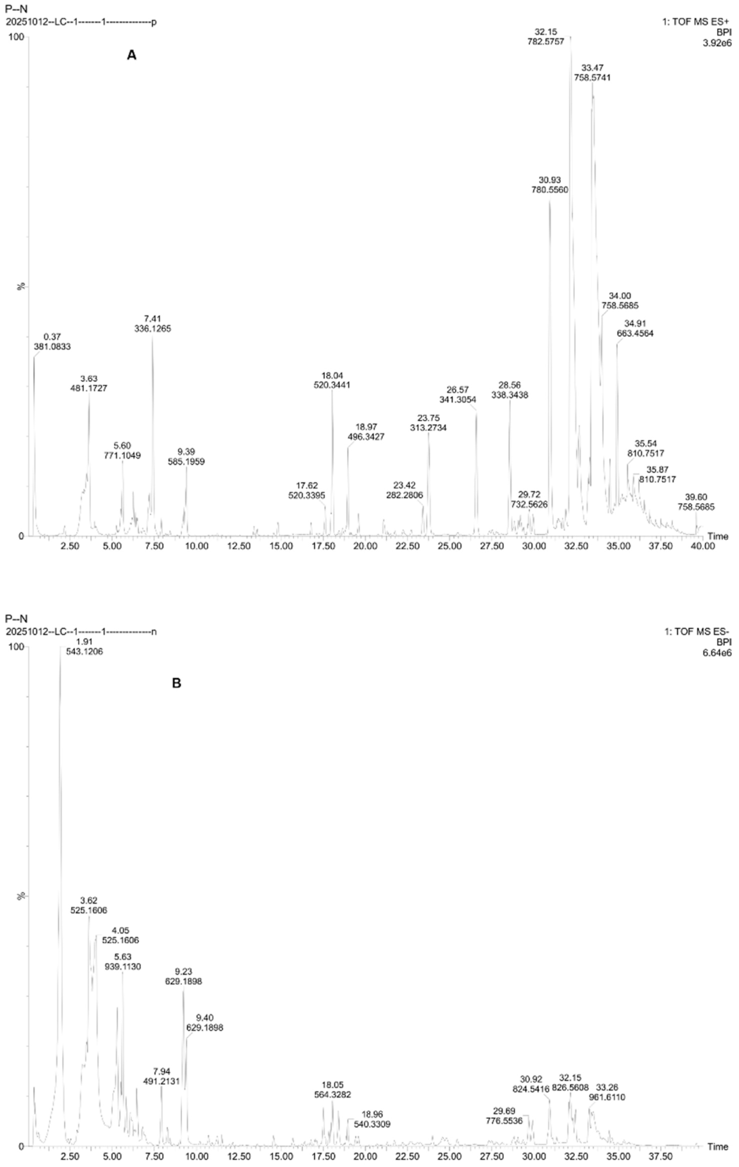

Figure S3. UPLC-QTOF-MS base peak ion chromatograms of the optimized ionic liquid-assisted sequential ultrasound–microwave extract of *Radix Paeoniae Alba*. (A) Positive ion mode; (B) negative ion mode. The chromatograms provide an overview of the optimized extract and should be interpreted as chemical-profile information rather than definitive structural identification.

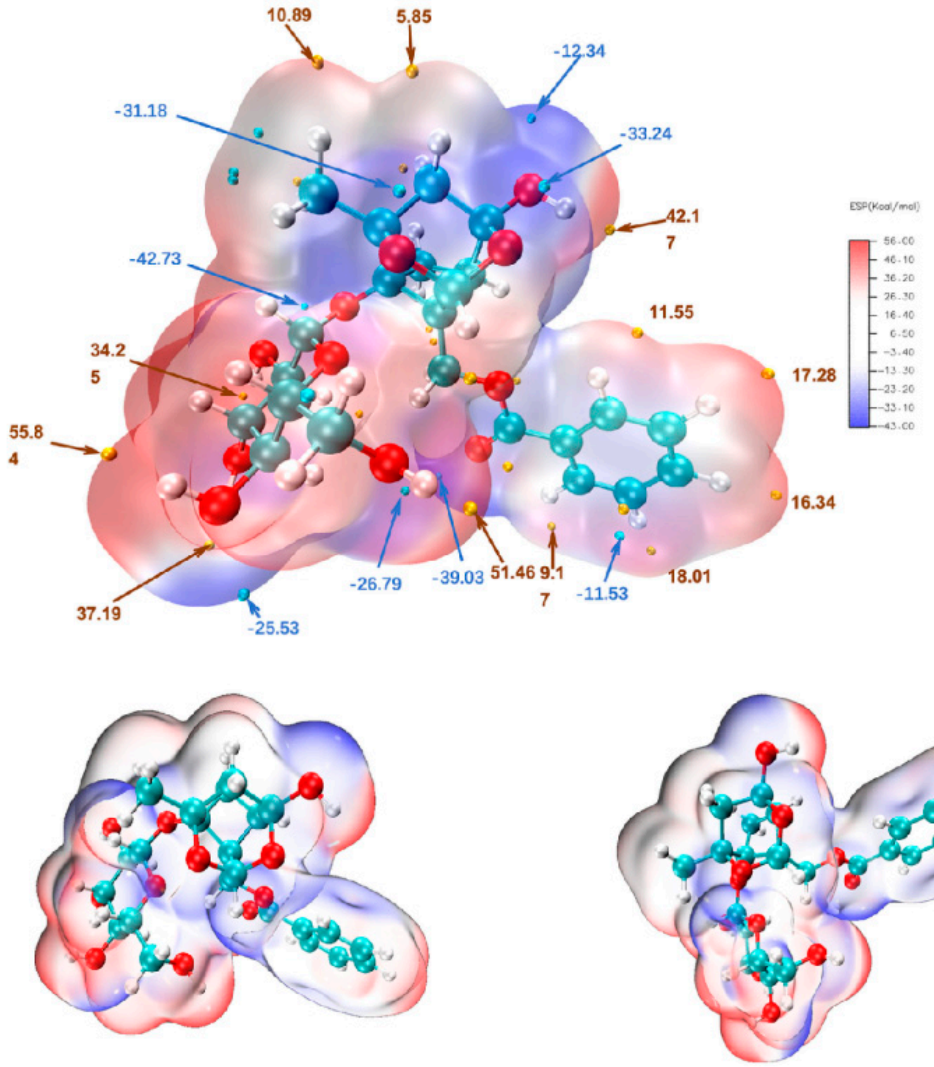

Figure S4. Additional electrostatic potential surface views of paeoniflorin. Red and blue regions represent negative and positive electrostatic potential regions, respectively. The electrostatic potential distribution indicates potential interaction sites around hydroxyl hydrogens and oxygen-containing groups, but does not by itself prove the formation of specific interactions during extraction.

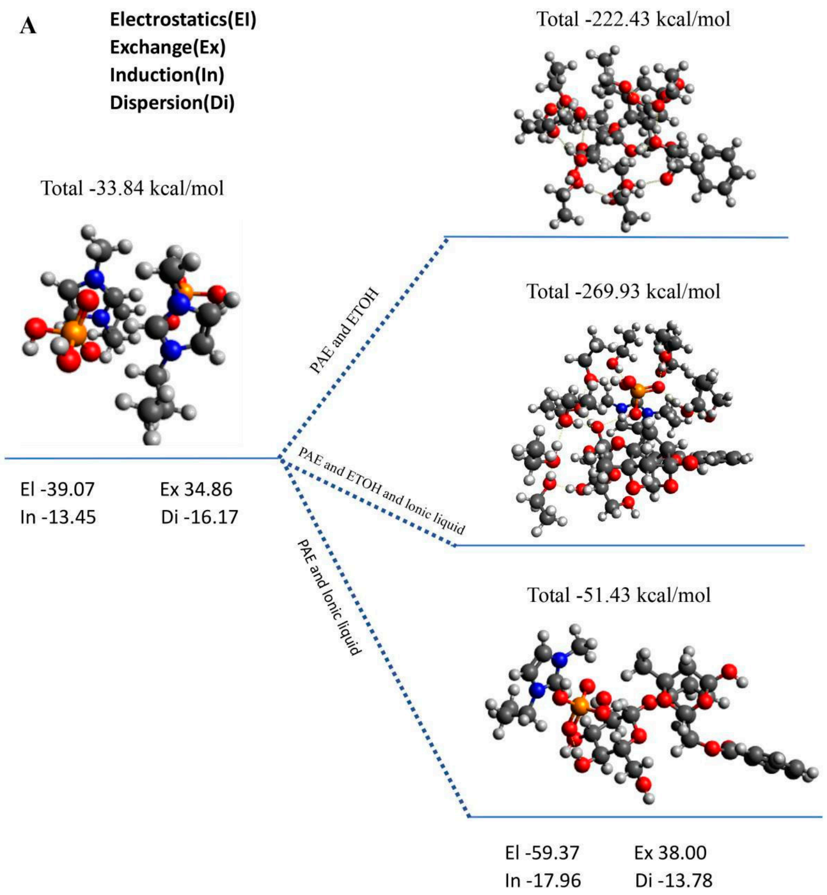

Figure S5. Additional SAPT energy decomposition results and selected optimized molecular models. The energy comparison includes simplified paeoniflorin-containing systems. In the simplified paeoniflorin–ionic liquid binary model, electrostatic interaction showed the largest attractive contribution, while induction and dispersion interactions also contributed to complex stabilization. These results are used as supportive molecular interaction information from simplified models rather than definitive proof of the complete extraction mechanism in the real plant matrix.

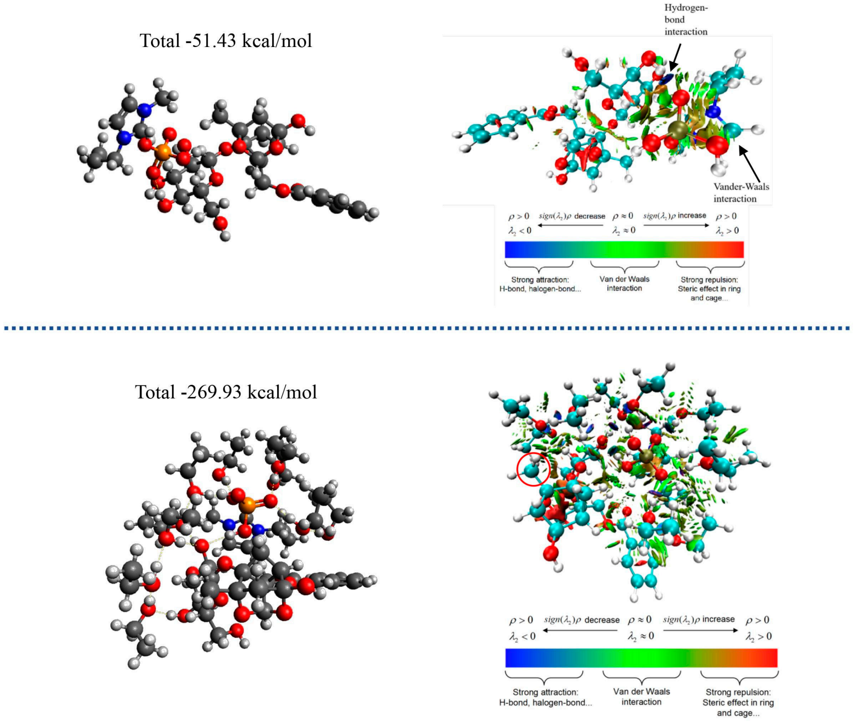

Figure S6. Additional non-covalent interaction plots of the simplified paeoniflorin–ionic liquid model. Green regions indicate van der Waals interactions, while blue regions indicate stronger attractive interactions such as hydrogen-bonding-related interactions. The NCI plots suggest possible interaction regions around hydroxyl and oxygen-containing groups, but the results are based on simplified optimized structures and do not fully reproduce solvent or plant-matrix effects.

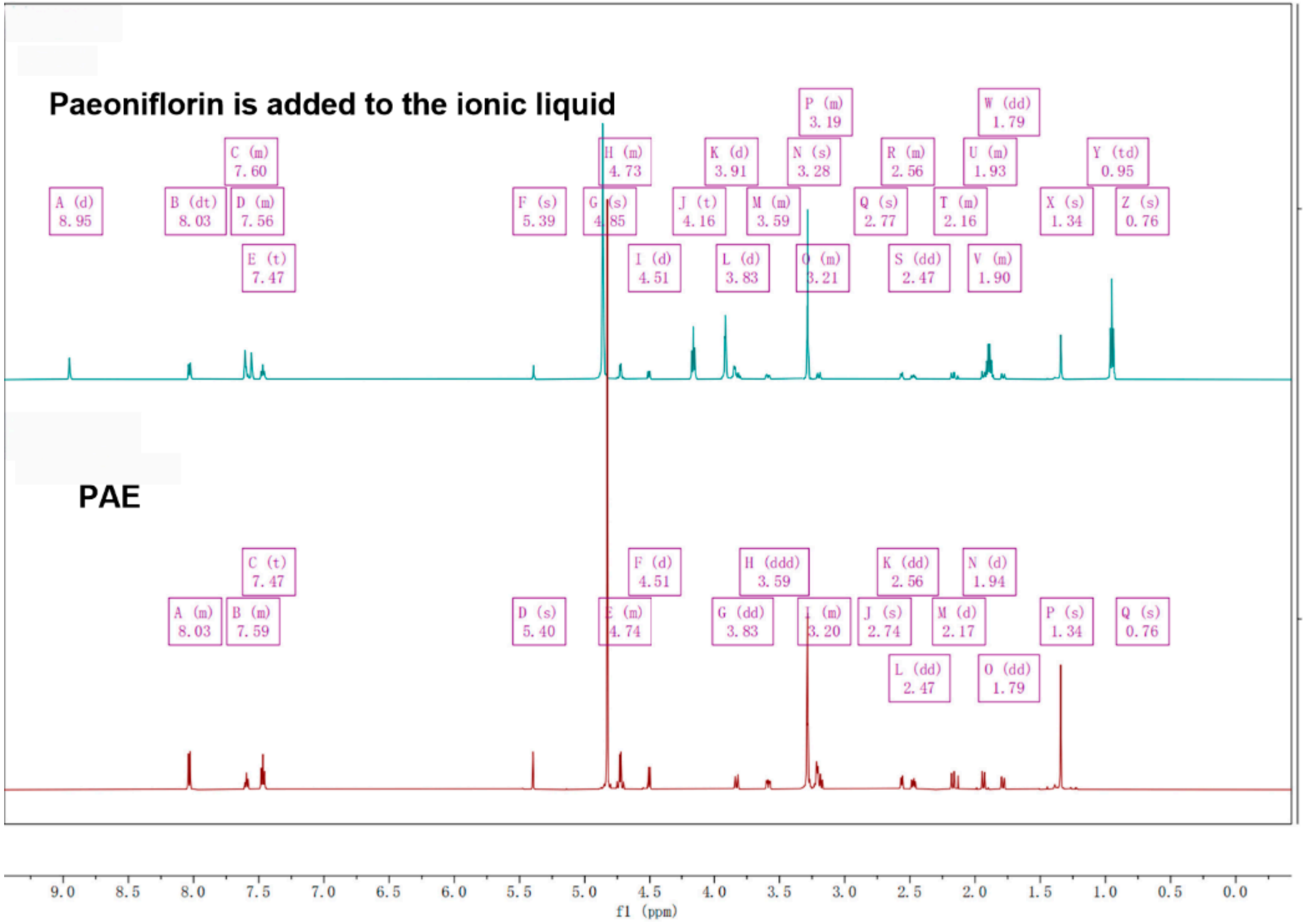

Figure S7. Full ^{1}H NMR spectra of paeoniflorin before and after ionic liquid addition. Changes in the 3.0–4.2 ppm region and peak-shape/chemical-shift perturbations were observed after addition of 1-propyl-3-methylimidazolium dihydrogen phosphate. Because complete signal assignment, two-dimensional NMR confirmation and concentration-dependent NMR analysis were not performed, the spectra are used only as supportive evidence for possible intermolecular interactions rather than definitive confirmation of a specific paeoniflorin–ionic liquid complex.

Supplementary Tables

Table S1. Detailed HPLC method validation data for oxypaeoniflorin, albiflorin and paeoniflorin.

Table S1-1. System suitability, linearity, LOD and LOQ.

Analyte	RT (min)	Resolution	Theoretical plates	Tailing factor	Calibration equation	Linear range (μg injected)	r	LOD(μg/mL)	LOQ(μg/mL)
Oxypaeoniflorin	6.351	1.599	6575	1.121	Y = 6E-10X + 2E-05	0.0391-2.5	0.9991	3.91	13.03

Albiflorin	12.13	13.56	8598	1.066	Y = 9E-10X + 1E-05	0.0391-2.5	0.9998	1.95	6.5
Paeoniflorin	14.99	4.903	9317	1.1	Y = 8E-10X + 2E-05	0.0391-2.5	0.9995	1.95	6.5

Abbreviations: RT, retention time; LOD, limit of detection; LOQ, limit of quantification. The linear range is expressed as injected amount, whereas LOD and LOQ are expressed as concentrations of analytical solutions. Calibration equations are presented as Y = injected amount and X = peak area according to the original source record. Full robustness evaluation was not claimed.

Table S1-2. Precision data based on repeated injections of the mixed standard solution.

Analyte	Peak area 1	Peak area 2	Peak area 3	Peak area 4	Peak area 5	RSD (%)
Oxypaeoniflorin	3750408	3702389	3749125	3764614	3898745	1.96
Albiflorin	2971774	2958367	2993737	3005570	2885363	1.59
Paeoniflorin	3383872	3388838	3387715	3414109	3339154	0.8

Table S1-3. Repeatability data based on independently prepared sample solutions.

Analyte	Peak area 1	Peak area 2	Peak area 3	Peak area 4	Peak area 5	RSD (%)
Oxypaeoniflorin	3435976	3346788	3312489	3326857	3331689	1.47
Albiflorin	2654216	2644571	2638583	2654036	2655596	0.28
Paeoniflorin	2918381	2904301	2907910	2899915	2900850	0.26

Table S1-4. Recovery data used for recovery-based trueness evaluation.

Analyte	Original sample amount (mg)	Recovered added amount used in calculation (mg)	Added standard amount (mg)	Recovery (%)	Mean recovery (%)	RSD (%)
Paeoniflorin	0.92	0.69	0.73	94.1	101.5	6.6
Paeoniflorin	0.92	0.85	0.8	106.8	101.5	6.6
Paeoniflorin	0.92	0.99	0.96	103.6	101.5	6.6
Oxypaeoniflorin	0.03	0.84	0.8	105.5	101.6	3.52
Oxypaeoniflorin	0.03	1.18	1.2	98.6	101.6	3.52
Oxypaeoniflorin	0.03	1.41	1.4	100.8	101.6	3.52
Albiflorin	0.28	0.79	0.8	98.34	101	2.89
Albiflorin	0.28	1.04	1	104.1	101	2.89
Albiflorin	0.28	1.21	1.2	100.6	101	2.89

RSD, relative standard deviation. Recovery (%) = recovered added amount / added standard amount × 100%. The column wording was adjusted to match the calculation reflected in the original source data; no new recovery values were generated.

Table S2. Ionic liquids used for anion and alkyl-chain-length screening.

Code	Ionic liquid / code used in manuscript	Source-record formula or abbreviation	Screening purpose	Supplier and purity/grade from available source records
L1	[Bmim]$C_5H_9NO_4$	[Bmim]$C_5H_9NO_4$	Anion screening	Shanghai Chengjie Chemical Co., Ltd.; 99% (specified in original source table)
L2	[Bmim]PF_6	[Bmim]PF_6	Anion screening	Shanghai Chengjie Chemical Co., Ltd.; 99% (specified in original source table)
L3	[Bmim]NO_3	[Bmim]NO_3	Anion screening	Shanghai Chengjie Chemical Co., Ltd.; 99% (specified in original source table)
L4	[Bmim]Br	[Bmim]Br	Anion screening	Shanghai Chengjie Chemical Co., Ltd.; 99% (specified in original source table)
L5	[Bmim]H_2PO_4	[Bmim]H_2PO_4	Anion screening	Shanghai Chengjie Chemical Co., Ltd.; 99% (specified in original source table)
T1	1-Propyl-3-methylimidazolium dihydrogen phosphate	$C_7H_{15}O_4N_2P$	Alkyl-chain-length screening	Shanghai Chengjie Chemical Co., Ltd.; 99% (specified in original source table)
T2	1-Butyl-3-methylimidazolium dihydrogen phosphate	$C_8H_{17}N_2O_4P$	Alkyl-chain-length screening	Shanghai Chengjie Chemical Co., Ltd.; 99% (specified in original source table)
T3	1-Pentyl-3-methylimidazolium dihydrogen phosphate	$C_9H_{19}O_4N_2P$	Alkyl-chain-length screening	Shanghai Chengjie Chemical Co., Ltd.; 99% (specified in original source table)
T4	1-Octyl-3-methylimidazolium dihydrogen phosphate	$C_{12}H_{25}O_4N_2P$	Alkyl-chain-length screening	Shanghai Chengjie Chemical Co., Ltd.; 99% (specified in original source table)
T5	1-Nonyl-3-methylimidazolium dihydrogen phosphate	$C_{13}H_{27}O_4N_2P$	Alkyl-chain-length screening	Shanghai Chengjie Chemical Co., Ltd.; 99% (specified in original source table)

Supplier and purity information were compiled from the available source records.

Table S3. Complete Box–Behnken design matrix and ANOVA results for response surface models.

Table S3-1. Additional model adequacy indices reported in the revised manuscript.

Response	R^2	Adjusted R^2	Predicted R^2	CV (%)	Adequate precision
Y1: Paeoniflorin content	0.9798	0.9539	0.7854	3.56	18.1431
Y2: Total marker monoterpene glycoside content	0.9845	0.9645	0.7979	3.02	20.9443

The models are used for optimization within the investigated factor ranges.

Table S3-2. Factors and coded levels used in the Box–Behnken design.

Factor	Variable	Unit	-1	0	+1
A	Solid-to-liquid ratio	mL/g (equivalent to 1:n g/mL)	20 (1:20)	25 (1:25)	30 (1:30)
B	Ionic liquid concentration	mol/L	0.05	0.1	0.15
C	Ultrasonic time	min	15	20	25

Table S3-3. Complete Box–Behnken design matrix and experimental responses.

Run	A actual (mL/g)	B actual (mol/L)	C actual (min)	A coded	B coded	C coded	Y1: Paeoniflorin content (mg/g)	Y2: Total marker monoterpene glycosides (mg/g)
1	20	0.05	20	-1	-1	0	16.28	19.58
2	30	0.05	20	1	-1	0	20.01	24.79
3	20	0.15	20	-1	1	0	23.04	27.66
4	30	0.15	20	1	1	0	25.76	30.99
5	20	0.1	15	-1	0	-1	19.17	23
6	30	0.1	15	1	0	-1	23.68	28.4
7	20	0.1	25	-1	0	1	22.88	27.41
8	30	0.1	25	1	0	1	25.98	31.09
9	25	0.05	15	0	-1	-1	20.77	24.91
10	25	0.15	15	0	1	-1	21.9	26.41
11	25	0.05	25	0	-1	1	20.44	24.69
12	25	0.15	25	0	1	1	27.01	31.94
13	25	0.1	20	0	0	0	29.15	34.39
14	25	0.1	20	0	0	0	29.4	34.52
15	25	0.1	20	0	0	0	27.62	33.36
16	25	0.1	20	0	0	0	28.79	34.71
17	25	0.1	20	0	0	0	28.48	34.34

Table S3-4. ANOVA for the fitted model of paeoniflorin content (Y1).

Source	df	Sum of squares	Mean square	F-value	p-value	Significance
Model	9	251	27.89	37.81	<0.0001	**
A: Solid-to-liquid ratio	1	24.71	24.71	33.5	0.0007	**
B: Ionic liquid concentration	1	51.06	51.06	69.21	<0.0001	**
C: Ultrasonic time	1	14.55	14.55	19.73	0.003	**
AB	1	0.255	0.255	0.3457	0.575	ns
AC	1	0.497	0.497	0.6737	0.4388	ns
BC	1	7.4	7.4	10.03	0.0158	*
A^2	1	51.84	51.84	70.28	<0.0001	**
B^2	1	64.26	64.26	87.1	<0.0001	**
C^2	1	21.34	21.34	28.93	0.001	**
Residual	7	5.16	0.7377			
Lack of fit	3	3.25	1.08	2.26	0.2233	ns
Pure error	4	1.91	0.4787			
Cor total	16	256.2				
Model statistics	R^2 = 0.9798; Adequate precision = 18.1431					

Table S3-5. ANOVA for the fitted model of total marker monoterpene glycoside content (Y2).

Source	df	Sum of squares	Mean square	F-value	p-value	Significance
Model	9	340.4	37.82	49.36	<0.0001	**
A: Solid-to-liquid ratio	1	38.81	38.81	50.65	0.0002	**

B: Ionic liquid concentration	1	66.3	66.3	86.54	<0.0001	**
C: Ultrasonic time	1	19.25	19.25	25.13	0.0015	**
AB	1	0.8836	0.8836	1.15	0.3185	ns
AC	1	0.7396	0.7396	0.9654	0.3586	ns
BC	1	8.27	8.27	10.79	0.0134	*
A^2	1	67.73	67.73	88.41	<0.0001	**
B^2	1	85.2	85.2	111.2	<0.0001	**
C^2	1	32.5	32.5	42.42	0.0003	**
Residual	7	5.36	0.7661			
Lack of fit	3	4.26	1.42	5.15	0.0737	ns
Pure error	4	1.1	0.2758			
Cor total	16	345.7				
Model statistics	R^2 = 0.9845; Adequate precision = 20.9443					

Table S3-6. Model-predicted optimum and verification results.

Item	Value
Predicted optimal condition	Solid-to-liquid ratio 1:25.79 g/mL, ionic liquid concentration 0.12 mol/L, ultrasonic time 21.60 min
Practical optimal condition	Solid-to-liquid ratio 1:26 g/mL, ionic liquid concentration 0.12 mol/L, ultrasonic time 22 min
Predicted Y1	29.6
Experimental Y1	29.12 mg/g, RSD 2.02%
Relative error for Y1	1.62%
Predicted Y2	35.28
Experimental Y2	34.98 mg/g, RSD 1.02%
Relative error for Y2	0.85%

Significance: **, $p < 0.01$; *, $p < 0.05$; ns, not significant. Y1, paeoniflorin content; Y2, total marker monoterpene glycoside content. Contents are expressed as mg/g dry material.

Table S4. Representative UPLC-QTOF-MS database-assisted candidate features in the optimized extract.

No.	Class	Putative assignment	Formula	Ion mode	RT (min)	Observed m/z	Observed ion	Mass error (ppm)	Evidence level / note
1	Monoterpene glycoside	Oxypaeoniflorin	$C_{23}H_{28}O_{12}$	ESI−	1.74	495.1497	[M−H]−	−2.1	Tentative; accurate mass + database match
2	Monoterpene glycoside derivative	Benzoylpaeoniflorin	$C_{30}H_{32}O_{12}$	ESI+	9.39	585.1965	[M+H]+	−0.2	Tentative; accurate mass + database match
3	Galloylated monoterpene glycoside	Galloylpaeoniflorin	$C_{30}H_{32}O_{15}$	ESI−	5.29	631.1673	[M−H]−	0.8	Tentative; accurate mass + database match
4	Galloylated monoterpene glycoside	Galloyloxypaeoniflorin	$C_{30}H_{32}O_{16}$	ESI−	5.46	647.1600	[M−H]−	−2.7	Tentative; accurate mass + database match
5	Monoterpene glycoside derivative	Oxidized benzoylpaeoniflorin	$C_{30}H_{32}O_{13}$	ESI−	9.21	599.1753	[M−H]−	−2.9	Tentative; accurate mass + database match
6	Phenolic acid	Gallic acid	$C_7H_6O_5$	ESI−	0.65	169.0143	[M−H]−	0.4	Tentative; accurate mass + database match
7	Galloylated sugar	Galloylglucose	$C_{13}H_{16}O_{10}$	ESI−	0.57	331.0672	[M−H]−	0.5	Tentative; accurate mass + database match
8	Gallotannin	1,6-Digalloyl-β-D-glucose	$C_{20}H_{20}O_{14}$	ESI−	2.27	483.0773	[M−H]−	−1.5	Tentative; accurate mass + database match
9	Gallotannin	1,2,6-Trigalloyl-β-D-glucose	$C_{27}H_{24}O_{18}$	ESI−	3.50	635.0873	[M−H]−	−2.7	Tentative; accurate mass + database match

10	Gallotannin	1,2,3,4,6-Pentagalloyl-β-D-glucose	$C_{41}H_{32}O_{26}$	ESI−	5.62	939.1115	[M−H]−	0.7	Tentative; accurate mass + database match

Abbreviations: RT, retention time; ESI, electrospray ionization. Candidate features are reported as tentative database-assisted annotations. Authentic-standard confirmation and raw MS/MS fragment/neutral-loss evidence were not available for all non-targeted candidates; therefore, no definitive structural identification is claimed.

AI-Assisted Graphical Preparation Statement

AI-assisted graphical preparation was used only for schematic layout preparation in the main manuscript figure. The scientific content, including computational results, SAPT energy components, NCI analysis and ^{1}H NMR evidence, was derived from the authors' original data and was checked by the authors. No AI tool was used to generate experimental data, analytical results or scientific conclusions.
